# Traumatic brain injury results in rapid pericyte loss followed by reactive pericytosis in the cerebral cortex

**DOI:** 10.1038/srep13497

**Published:** 2015-09-03

**Authors:** Christoph M. Zehendner, Anne Sebastiani, André Hugonnet, Florian Bischoff, Heiko J. Luhmann, Serge C. Thal

**Affiliations:** 1 Institute of Physiology, University Medical Center of the Johannes Gutenberg-University, Mainz, Germany; 2 Department of Anesthesiology, University Medical Center of the Johannes Gutenberg-University, Mainz, Germany

## Abstract

Accumulating evidence suggests a pivotal role of PDGFRß positive cells, a specific marker for central nervous system (CNS) pericytes, in tissue scarring. Identification of cells that contribute to tissue reorganization in the CNS upon injury is a crucial step to develop novel treatment strategies in regenerative medicine. It has been shown that pericytes contribute to scar formation in the spinal cord. It is further known that ischemia initially triggers pericyte loss *in vivo*, whilst brain trauma is capable of inducing pericyte detachment from cerebral vessels. These data point towards a significant role of pericytes in CNS injury. The temporal and spatial dynamics of PDGFRß cells and their responses in traumatic brain injury are poorly understood. Here we show that PDGFRß positive cells initially decline in the acute phase following experimental traumatic brain injury. However, PDGFRß positive cells increase significantly in the trauma zone days after brain injury. Using various pericyte markers we identify these cells to be pericytes that are demarcated by reactive gliosis. Our data indicate that brain trauma causes a biphasic response of pericytes in the early phase of brain trauma that may be of relevance for the understanding of pathological cellular responses in traumatic brain injury.

Pericytes play a pivotal role in a number of physiological and pathophysiological processes. They are of tremendous importance for vessel stability in various organ systems, e.g. the blood brain barrier (BBB) and in coronary vessels^1^,^2^, but also in tumor growth and vascularization^3^. Importantly, pericytes contribute to the formation of the neurovascular unit, which is comprised of brain endothelial cells, pericytes, astrocytes, neurons, and glial cells^4^. Here, they are important in the regulation and development of the BBB^5^ and contribute to its proper function^6^. A loss of CNS pericytes induces BBB breakdown, neuroinflammation^6^, and CNS aneurysm formation^7^. In addition, pericytes are capable of regulating capillary diameter and microvascular perfusion within the CNS^6^,^8^,^9^.

In disease states such as cerebral ischemia, it has been shown that hypoxia causes pericyte death and persistent CNS microvascular obstruction^8^,^10^. We have previously shown a loss of pericytes in an *in vitro* preparation of the developing cerebral cortex upon ischemic and inflammatory stimuli^11^.

On the other hand, abnormal pericyte proliferation has been reported upon pathophysiological stimuli such as spine trauma and it is known that pericytes contribute to organ scar formation and tissue fibrosis^12^,^13^,^14^. Pericytes are further a promising cellular source for novel cellular therapy concepts. For example human pericytes have been reprogrammed recently into induced neuronal cells^15^,^16^. More than a decade ago, Dore-Duffy *et al.* have demonstrated that pericytes are capable of migrating away from the vessel wall after traumatic brain injury (TBI)^32^. Experimental data from a murine cerebral ischemia model^17^,^18^ have shown that platelet-derived growth factor receptor beta (PDGFRß), a highly specific marker for pericytes within the CNS^19^, has an important role in ischemic tissue responses upon injury. Fernández-Klett *et al.* have observed a significant increase in PDGFRß cellular mass formation within the ischemic core in a murine middle cerebral artery occlusion (MCAO) starting at days 3–5 after ischemic insult^17^. A better characterization of PDGFRß positive cell responses in TBI may therefore be of clinical importance in order to better understand and modulate TBI pathophysiology.

We were therefore interested in the temporal and spatial dynamics of PDGFRß positive cell formation following cortical controlled impact (CCI).

## Material and Methods

### Induction of CCI and ethical statement

**Figure 1:**
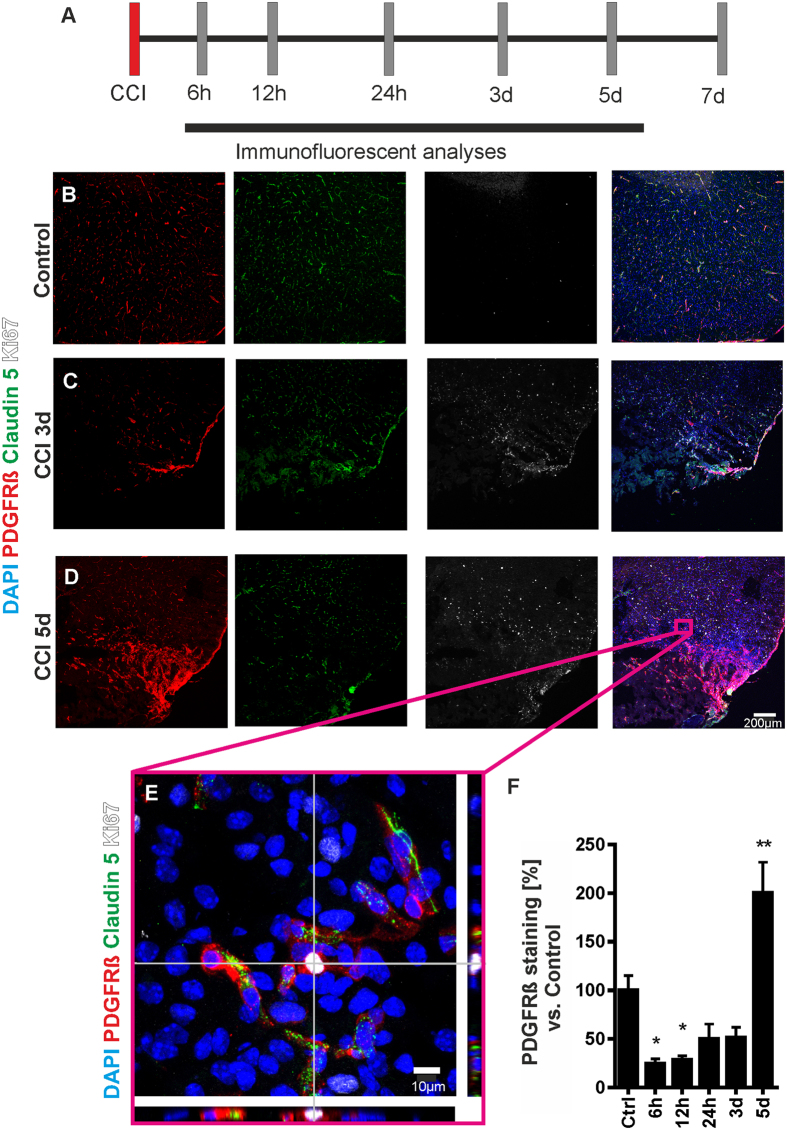
CCI results in pericontusional pericyte proliferation. Tissue was collected for immunohistochemical and mRNA analyses at varying time points after induction of CCI (**A**). Under physiological conditions pericytes were located in a typical perivascular manner within the cerebral cortex (Claudin 5: green; PDGFRß: red). Only few cells were positive for Ki67, a marker for cell proliferation (**B**). Three days after CCI PDGFRß positive cells at the CCI border were associated with a strong increase of Ki67 immunoreactivity (**C**). Five days after CCI a prominent increase of PDGFRß expressing cells was detected (**D**). Panel **E** shows an orthogonal section view of a pericontusional pericyte positive for Ki67 that is not in contact with a microvessel. Quantification of PDGFRß staining demonstrates an initial loss of PDGFRß that is followed by a significant increase of PDGFRß 5 days after CCI (**F**). N = 5–6 animals per group; *P < 0.05; **P < 0.01.

C57/Bl6 mice were subjected towards CCI as described in detail elsewhere^20^. In order to detect CCI-induced cellular alterations, animals were compared with naïve mice in which the physiological arrangement of the neurovascular unit is preserved. In brief mice were anesthetized with isoflurane. Body temperature was monitored and maintained at 37 °C with the help of a heating pad throughout the experiment. For CCI a small skin incision above the skull was made and a burr hole was drilled. Then a precisely controlled pneumatic insult was applied above the right cortex with a custom made impactor (Supplementary Figure S1, diameter 3 mm, penetration depth 1.5 mm, velocity 8 m/s). After this procedure animals were allowed to recover and were clinically monitored regularly. Mice were sacrificed at several varying time points following injury and brains were collected for immunohistochemical processing or mRNA analyses (Fig. 1A). All experimental procedures were approved by the local ethical committee of the University Medical Center of the Johannes Gutenberg-University Mainz. The study protocol was approved by the Landesuntersuchungsamt RLP (protocol number: G12-1-010). All efforts were made to minimize the number of animals used and their suffering. European guidelines on the handling of animals were followed.

### Immunohistochemistry

Immunoreactive stainings were performed as documented before^11^,^20^. In brief brains were snap frozen in dry ice and cryosliced at 20 μm thickness (at Bregma −1 mm according to mouse brain atlas, mbl.org) with a cryostat (CryoStar™ NX70 Kryostat, Fischer Scientific). Care was taken to acquire sections from the same coordinates. Following cryoslicing probes were fixed in ice cold acetone for 5 minutes. Then tissue was further processed for immunostaining.Brain slices were blocked and permeabilized in 0.01 mol/l PBS containing 7% normal goat serum (Jackson Immuno Research Dianova, Hamburg, Germany) and 0.3% triton (Sigma-Aldrich Steinheim, Germany) for 2 hours. Then probes were incubated with primary antibodies in 2% bovine serum albumin (Dianova) with 0.05% azide and 0.1% Triton in 0.01 mol/l PBS overnight at room temperature. Subsequently probes were carefully washed with 0.01 mol/l PBS and then incubated with secondary antibodies in 2% bovine serum albumin for 2 hours at room temperature. After washing in 0.01 mol/l PBS sections were embedded in Fluoromount (Southern Biotech, Birmingham, AL, USA).

Table 1 and Table 2 give an overview on primary and secondary antibodies and dilutions used in this study.

### Confocal imaging

Imaging was performed with a Leica SP5 confocal microscope using Leica Application Suite Advanced Fluorescence (Leica Microsystems). Excitations wavelengths were 405, 488, 561 and 633 nm. Care was taken to perform imaging with same parameters regarding exposure time and laser intensity for all samples.

### Image analysis

Quantitative analyses were performed with NIH “Fiji is just Image J” (http://fiji.sc/Fiji). In order to quantify PDGFRß positive stained cells, Z-stacks were collected from each animal in the pericontusional zone (yellow region in Supplementary Figure S1). Z-stacks were Z projected at maximum intensity and binary images were created. Finally average gray scale intensity was acquired by executing the Fiji command “measure”. Details on the calculation of gray scale values by Fiji are documented at the NIH webpage: http://rsb.info.nih.gov/ij/docs/menus/analyze.html. Animated 3 dimensional reconstructions from acquired z-stacks were created using IMARIS (Bitplane, Switzerland).

### Gene expression (mRNA) analysis

For time series analysis tissue preparation was performed as follows: Brains were quickly removed and placed in a cooled brain matrix (Zivic Instruments, Pittsburgh, USA). Perilesional injured brain tissue was immediately dissected, rapidly frozen in liquid nitrogen and stored at −80 °C until processing. Quantification of mRNA was performed by real-time polymerase chain reaction as described previously^21^. Absolute copy numbers of target genes were normalized against cyclophilin A (PPIA) as housekeeping gene^22^. Applied primers and probes are shown in Table 3. Same amounts of cDNA were amplified in duplicates using ABsolute™ Fast qPCR SYBR Green Mix (Thermo Fisher Scientific, Dreieich, Germany) for PPIA, PDGFRß, NG2, and Desmin according to the manufacturer’s instructions.

### Statistics

For statistical analyses GraphPad Prism version 6.0 for Windows was used. All datasets were checked for normalization using the D’Agostino and Pearson method^23^. All datasets following a Gaussian distribution were analyzed using ONE WAY ANOVA followed by Dunnett’s test for multiple comparisons. Non-parametric data were analyzed with a Kruskal Wallis Test followed by a Dunns multiple comparison test. Differences were considered to be significant at alpha <0.05. Data are displayed as mean ± standard error of the mean.

## Results

### Impact of CCI on PDGFRß staining in the pericontusional zone

In the cerebral cortex of naïve control animals we found a physiological perivascular arrangement of PDGFRß positive cells. Under physiological conditions, only few cells were positive for the proliferation marker Ki67 (Fig. 1B). Three days after CCI the expression of the proliferation marker Ki67 increased within the pericontusional zone (Fig. 1C). Five days after CCI a significant cellular mass formation positive for the pericyte marker PDGFRß and Ki67 became apparent at the site of the lesion (Fig. 1D, inset E). High power imaging revealed PDGFRß positive cells that were immunoreactive for Ki67 (Fig. 1D). Many of these PDGFRß positive cells were not in contact with microvessels as documented in co-stainings with the microvascular marker Cl-5, which has been shown to be a specific marker for brain endothelial cells in brain trauma (Fig. 1E, Supplementary Movie 1)^20^,^24^.

Quantitative analyses revealed a significant decrease in PDGFRß staining 6–12 hours following CCI in the pericontusional zone (Fig. 1F). At 24 hours and 3 days post CCI no significant difference in PDGFRß positive cells could be detected when compared with naïve controls (Fig. 1F). However, on day 5 there was a significant increase of PDGFRß positive cells compared with controls (Fig. 1F).

**Figure 2:**
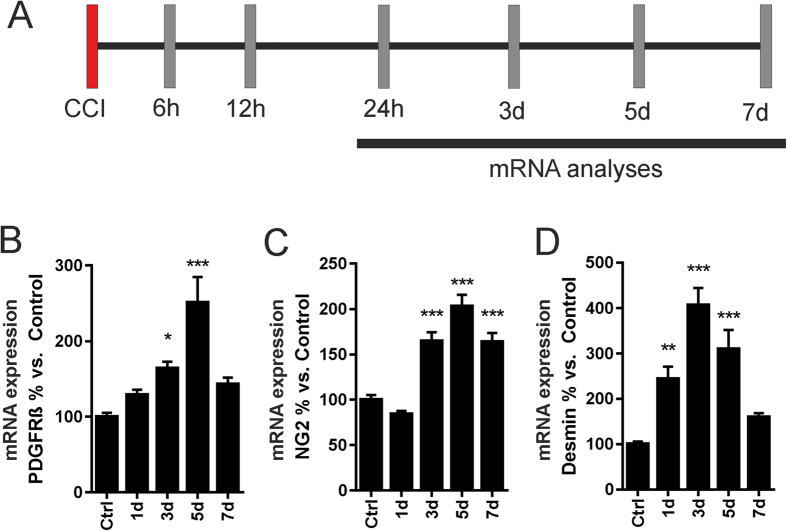
Pericyte differentiation markers are upregulated in a time dependent manner following CCI. The pericyte expression markers PDGFRß, NG2, and Desmin were significantly upregulated following CCI. mRNA analyses were carried out on days 1, 3, 5 and 7 following TBI (**A**). PDGFRß mRNA levels were found to increase significantly, starting on day 3 after CCI with a peak at day 5 (**B**). The pericyte marker NG2 was also upregulated upon CCI from days 3–7 (**C**). Here, maximum NG2 mRNA levels were detected 5 days after brain trauma induction. The pericyte marker Desmin was already significantly upregulated on day 1, reaching its maximum expression levels on day 3 after CCI (**D**). N = 8–10 animals per group; *P < 0.05; **P < 0.01; ***P < 0.001.

Since PDGFRß is a specific marker for pericytes within the CNS, we further analyzed mRNA levels of pericyte markers on day 1, 3, 5 and 7 after CCI induction (Fig. 2A). Here we found that mRNA levels of the pericyte markers PDGFRß and NG2 were significantly upregulated after CCI with a peak on day 5 (Fig. 2B,C). The pericyte marker Desmin was already significantly upregulated on day 1 following CCI reaching its maximum mRNA levels on day 3 (Fig. 2D).

### PDGFRß positive cells in CCI are surrounded by a basement membrane

**Figure 3:**
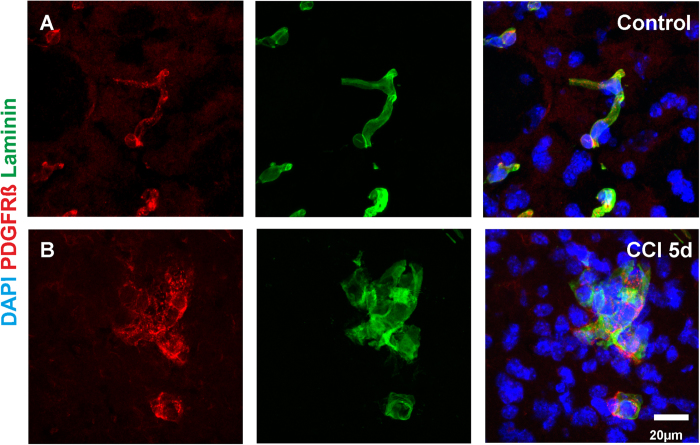
Pericytes in control and CCI conditions are sheathed by a basement membrane. In order to verify the pericyte identity of PDGFRß positive cells, we performed pan-laminin co-stainings. In both, control (**A**) and CCI animals (**B**), PDGFRß positive cells were found to be sheathed by a basement membrane, a hallmark to identify pericytes.

We and others have previously documented that PDGFRß is a specific marker for pericytes in the CNS^11^,^19^,^25^. Another property of pericytes is the presence of a basement membrane (BM) that maintains several laminin isoforms^11^,^26^. Association with a BM is an important criterion to identify pericytes^27^. By using a pan-laminin antibody, that recognizes several laminin isoforms, we observed that pericytes in intact cortices (Fig. 3A, Supplementary movie 2), as well as PDGFR beta positive cells in the pericontusional tissue after CCI are surrounded by a BM (Fig. 3B, Supplementary movie 3). Interestingly, pericytes in the pericontusional zone display an amoeboid like morphology that is significantly different from the perivascular morphology of pericytes in physiological conditions (Fig. 3A,B, Supplementary movies 2–5).

### PDGFRß positive cells express various pericyte differentiation markers

**Figure 4:**
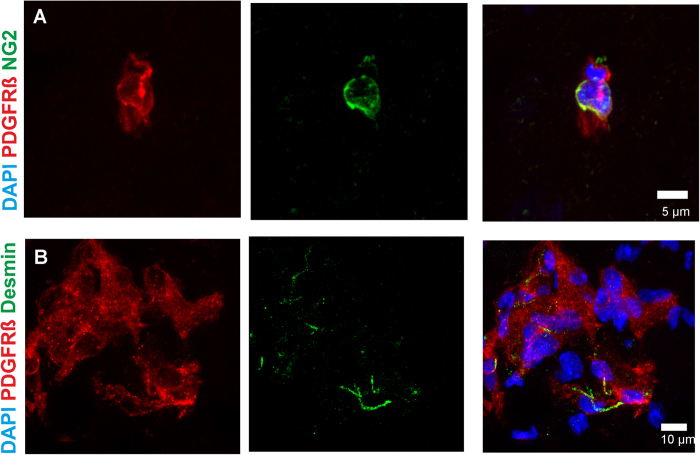
PGDFRß cells in the pericontusional zone express the pericyte markers NG2 and Desmin. Co-staining with the pericyte marker NG2 demonstrates that PDGFRß positive cells are immunoreactive for NG2 at day 5 after brain trauma (**A**). Note the typical perinuclear expression of NG2. In addition we found that some PDGFRß positive cells expressed the pericyte marker Desmin (**B**). Note that NG2 is a perinuclear protein whilst Desmin is an intracellular filament protein. Therefore both markers do not totally overlap with PDGFRß due to their different subcellular localization.

In order to further characterize PDGFRß positive cells in the pericontusional CCI zone, we performed co-stainings with a set of established pericyte differentiation markers such as NG2, Desmin, and PDGFRß (Fig. 4). Here we found that PDGFRß co-localized with NG2 and partly with Desmin, indicating that PDGFRß cells were indeed pericytes (Fig. 4A,B). However there is not a 100 percent overlap between all markers. This is due to the different cellular localization of the respective proteins. PDGFRß is a transmembraneous tyrosine kinase, whilst Desmin is an intracellular intermediate filament protein^28^. NG2 is mostly expressed in a perinuclear manner in mature CNS pericytes that is also reflected by the immunofluorecent image in Fig. 4. However, we would like to point out that this discrepancy of marker overlap might also be partly due to a lack of specificity of these cellular markers.

### Reactive glia demarcates PDGFRß cell formation

**Figure 5:**
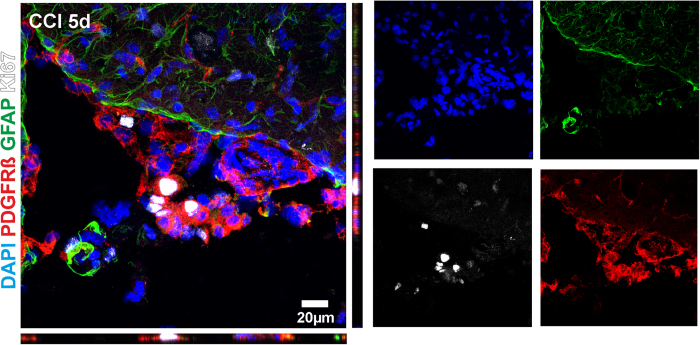
Reactive astrocytosis demarcates proliferative PDGFRß cellular mass formation. Immunofluorescent confocal analyses revealed that PDGFRß cells 5 days after CCI were a distinct cell population and different from reactive astrocytes. Co-stains for GFAP, a marker for reactive astrogliosis, and PDGFRß demonstrate that reactive astrocytes demarcate a large number of proliferating PDGFRß positive cells. Note the high expression level of Ki67 in PDGFRß positive cells.

It is well established that GFAP expressing reactive astrocytes are a major contributor to scar formation^13^,^29^ in the CNS. It has been recently documented in a model of murine ischemic stroke that proliferation of PDGFRß cells occurs in the ischemic core, which is demarcated by reactive astrogliosis^25^. In order to evaluate whether the observed PDGFRß cell proliferation is demarcated in a similar manner by reactive astrogliosis in CCI, we performed co-stainings with PDGFRß and the reactive astrocyte marker GFAP following CCI. In line with the literature we found that a high number of reactive astrocytes were present in the pericontusional zone. Similar to the results of Fernandez-Klett *et al.* in cerebral ischemia^25^, we found that Ki67 positive pericytes were demarcated by reactive astroglia 5 days after CCI (Fig. 5). In addition, PDGFRß and GFAP co-staining gave no evidence for an overlap of both immunohistochemical markers, indicating that PDGFRß and GFAP positive cells are distinct cell types.

## Discussion

Here we show in a murine model of CCI that pericytes undergo a biphasic cell response within the first 5 days after TBI.

A rapid loss of PDGFRß cells within the first 12 hours was followed by a significant increase of PDGFRß positive cells on day 5 after TBI. In our study PDGFRß immunoreactive cells expressed several pericyte markers such as Desmin and NG2 on protein level, pointing towards a pericyte identity. In addition, our qRT-PCR results on the increase of the pericyte markers NG2, Desmin and PDGFRß following TBI further support the immunofluorescence data.

However, the morphology of PDGFRß cells in CCI was different compared with physiological conditions. PDGFRß cells in the pericontusional TBI zone were amoeboid shaped and not necessarily in vasular contact. Co-stainings with GFAP revealed that PDGFRß cell mass formation was demarcated by reactive glia. Further these immunohistochemical results indicate that PDGFRß cells do not express GFAP, indicating they are not a subpopulation of reactive astrocytes.

Three to five days following brain trauma, Ki67 was found to be expressed by PDGFRß positive cells, which was not the case in physiological conditions. In line with our data on PDGFRß staining on day 5 after TBI, Fernández-Klett *et al.* have shown that PDGFRß positive cells significantly increase in a model of middle cerebral artery occlusion followed by reperfusion 5 days after stroke in mice^25^. In addition, the authors from that study also observed a demarcation of PDGFRß cell mass formation by reactive glia, which was also the case in our experimental study.

Interestingly, an enhanced cell proliferation was absent 2 weeks after brain trauma insult in a report by Chen *et al.*^30^. In our study we investigated proliferation for up to 5 days following trauma. Therefore our results may implicate that Ki67 is only transiently increased. However the trauma model used by Chen *et al.*^30^ is different to that used in our present study and the investigated species was rat whilst we examined C57/Bl6 mice. Our finding that pericytes play an important role in cellular brain injury responses is in line with data from Birbair *et al.* who have recently shown that 14 days after brain trauma, pericytes contribute to scar formation in the cortex^14^. However, in the study by Birbair *et al.* there was a mismatch in NG2 and PDGFRß expression 14 days after injury. Since we observed a colocalization of NG2 and PDGFRß immunoreactivity up to 5 days after injury, we speculate that the expression of both pericyte markers may undergo regulatory changes over longer time periods following TBI.

PDGFRß ablation experiments have shown that loss of PDGFRß induces pericyte loss, leading to an increase of BBB permeability, which results in extravasation of acute phase proteins into the CNS parenchyma that enhance secondary brain injury due to inflammation^6^,^31^. Therefore, therapeutic strategies to modulate PDGFRß related cell responses upon CNS injury may help to improve clinical outcome. We speculate that the observed biphasic response of PDGFRß positive cells within the first 5 days of TBI may present a promising target to modulate clinical complications such as evolution of brain edema or improve nerve fibre regeneration^13^,^31^.

In summary, our *in vivo* study identifies CNS pericytes as a cell population that declines in the acute phase of TBI, which is followed by reactive pericytosis on day 5 following cortical brain trauma. More research needs to be done in order to unravel the pathophysiological relevance of the observed biphasic pattern of PDGFRß cell dynamics.

## Additional Information

**How to cite this article**: Zehendner, C. M. *et al.* Traumatic brain injury results in rapid pericyte loss followed by reactive pericytosis in the cerebral cortex. *Sci. Rep.* **5**, 13497; doi: 10.1038/srep13497 (2015).

## Supplementary Material

Supplementary Information

Supplementary Movie 1

Supplementary Movie 2

Supplementary Movie 3

Supplementary Movie 4

Supplementary Movie 5

## Figures and Tables

**Table 1:** Primary antibodies and their respective application.

Primary antibodies	Application
anti-claudin 5 (Life technologies #35-2500), 1:50	Brain endothelial cell marker and tight junction protein
anti-GFAP (Dakocytomation, Z 0334), 1:200	Marker of reactive astrocytes
anti-PDGFRß (Neuromics, GT 15065), 1:200	Marker of pericytes
anti-Ki67 (ab 15580 Abcam), 1:200	Cell proliferation marker
DAPI (Sigma, 32670), 0.5 μg/ml	Cell nuclei staining
anti-Desmin (4024 Cell Signaling), 1:100	Pericyte marker
anti-pan-Laminin (ab7463, Abcam), 1:1000	Staining for basement membrane and laminins
anti-NG2 (AB5320: Merck Millipore), 1:200	Staining for pericyte marker NG2

**Table 2:** Secondary Antibodies.

Secondary antibodies	related primary antibodies
Cy3 (705-165-147, Jackson, Dianova), 1:200	PDGFRß
DyLight 488 (A90-337D2, Biomol), 1:200	Claudin 5, GFAP
Alexa Fluor 647 (711-605-152, Jackson, Dianova), 1:200	Ki67, Laminin, NG2

**Table 3:** Primer sequences.

PCR Assay (amplicon size,annealing temp)	Oligonucleotide Sequence (5′-3′)	Gene Bank No.
Cyclophilin A (PPIA, 146 bp, 58 °C)	Forw: 5′-GCGTCTSCTTCGAGCTGTT-3′Rev: 5′-RAAGTCACCACCCTGGCA-3′	NM_008907
PDGFRß (152 bp, 58 °C)	Forw: 5′-AGGAGTGATACCAGCTTTAGTCC-3′Rev: 5′-CCGAGCAGGTCAGAACAAAGG-3′	NM_001146268
NG2 (Cspg4, 132 bp, 58 °C)	Forw: 5′-GGGCTGTGCTGTCTGTTGA-3′Rev: 5′-TGATTCCCTTCAGGTAAGGCA-3′	NM_139001
Desmin (243 bp, 58 °C)	Forw: 5′-CCTGGAGCGCAGAATCGAAT-3′Rev: 5′-TGAGTCAAGTCTGAAACCTTGGA-3′	NM_010043
