# Supplementary material for: Traumatic brain injury results in rapid pericyte loss followed by reactive pericytosis in the cerebral cortex
Source: Sci Rep. 2015 Sep 3;5:13497. doi: 10.1038/srep13497 (PMC4558600; doi:10.1038/srep13497)
Supplement: Supplementary Information [file srep13497-s1.pdf]

Supplementary Material

Traumatic brain injury results in rapid pericyte loss followed by reactive pericytosis in the cerebral cortex

Christoph M. Zehendner[1,2§], Anne Sebastiani[3], André Hugonnet[3], Florian Bischoff[1], Heiko J. Luhmann[1], Serge C. Thal[3§]

1 Current address: ZIM III, Department of Cardiology, Institute for Cardiovascular Regeneration, Goethe University Frankfurt, Germany
2 Institute of Physiology, University Medical Center of the Johannes Gutenberg-University, Mainz, Germany
3 Department of Anesthesiology, University Medical Center of the Johannes Gutenberg-University, Mainz, Germany

[§] Corresponding author contact:

Christoph M. Zehendner, MD
ZIM III, Department of Cardiology
Institute for Cardiovascular Regeneration, Center of Molecular Medicine, Goethe University
Theodor Stern Kai 7
60590 Frankfurt am Main, Germany
Phone: 5
Fax: +49-69-6301-83462
Email: zehendner@med.uni-frankfurt.de; Christoph.Zehendner@gmail.com

Serge C. Thal, MD
Department of Anesthesiology, University Medical Center of the Johannes Gutenberg-University
Langenbeckstrasse 1
55131 Mainz, Germany
Phone: +49-6131-17-6754
Fax: +49-6131-17-5599
Email: thal@uni-mainz.de

Supplementary figures and movies

Supplementary Figure S1

Anatomic overview of cryosectioning, controlled cortical impact (CCI) localization and confocal image acquisition

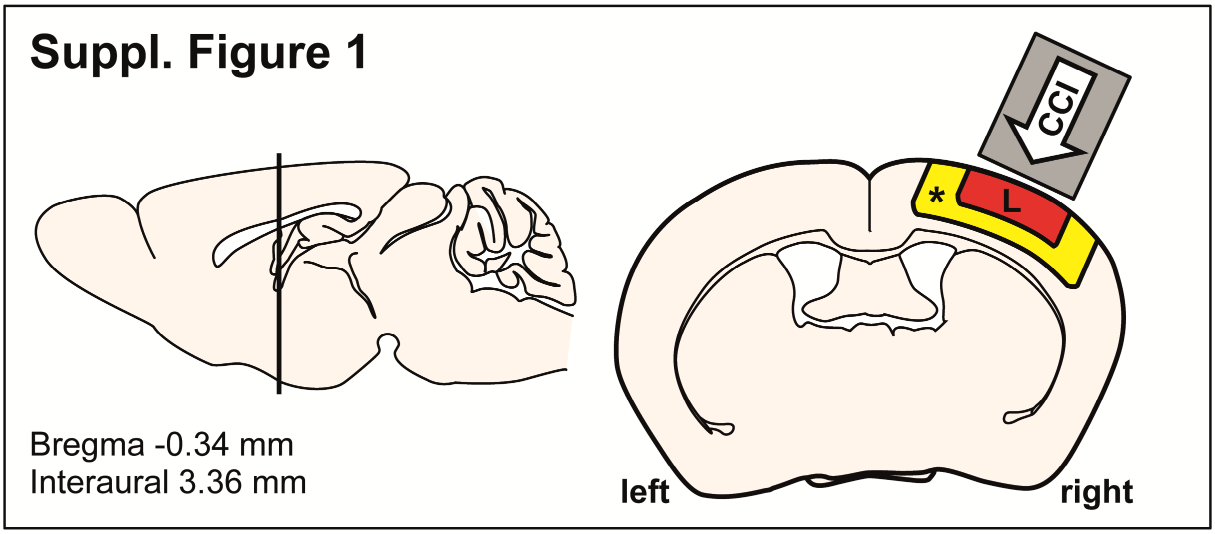

Anatomic overview of cryosectioning, controlled cortical impact (CCI) localization and confocal image acquisition. L marks the induction of the primary lesion (highlighted in red). Asterisk indicates imaging area (highlighted in yellow). Arrowhead depicts lesion induction (CCI).

Supplementary movie S1

Pericyte detachment from cerebral vessels in CCI.

An animated 3D reconstruction demonstrates PDGFRß staining (red) in the pericontusional zone and brain vessel organization (Claudin 5 in green). Note that several PDGFRß positive cells are not in vascular contact in the pericontusional zone. Note that with increasing distance from the primary CCI lesion, pericyte detachment from vessels is less visible.

Supplementary movie S2

PDGFRß positive cells are ensheathed by a basement membrane in physiological conditions

PDGFRß positive cells are sheathed by a basement membrane in physiological conditions. Movie S2 depicts a 3D reconstruction of a PDGFRß positive pericyte in the cortical layer of a naïve control animal (also shown in Figure 3A). Note that the PDGFRß stain (red) is surrounded by laminin (pan-laminin antibody, green). Nuclei are labelled in blue (Hoechst).

Supplementary movie S3

PDGFRß positive cells are ensheathed by a basement membrane in TBI conditions

Movie S3 demonstrates that in brain trauma, PDGFRß positive cells (red) were sheathed by a basement membrane (labelled in green, also shown in Figure 3B). Cellular nuclei are depicted in blue (Hoechst).

Supplementary movie S4

<u>Physiological pericyte morphology</u>

Movie S4 demonstrates the physiological morphology of a pericyte, for better visualization, only the blue (Hoechst, cell nuclei) and red (PDGFRß) channel from movie S2 are shown.

Supplementary movie S5

<u>Pericytes display an amoeboid shape in TBI conditions</u>

Movie S5 depicts the amoeboid shape of pericytes in the pericontusional zone after experimental TBI. For better visualization, only cell nuclei (blue) and PDGFRß (red) are shown.
